# Selective and prolonged attention to emotional scenes in humans and bonobos

**DOI:** 10.1098/rspb.2024.0433

**Published:** 2024-08-07

**Authors:** Evy van Berlo, Tom S. Roth, Yena Kim, Mariska E. Kret

**Affiliations:** ^1^ Institute of Psychology, Cognitive Psychology Unit, Leiden University, Leiden, The Netherlands; ^2^ Leiden Institute for Brain and Cognition, Leiden, The Netherlands; ^3^ Institute for Biodiversity and Ecosystem Dynamics, Evolutionary and Population Biology, University of Amsterdam, Amsterdam, The Netherlands; ^4^ Department of Biology, Animal Behaviour and Cognition, Utrecht University, Utrecht, The Netherlands

**Keywords:** affect, emotion, comparative, attention bias, eye-tracking

## Abstract

Perceiving emotions in others is at the foundation of higher-order social cognition. The importance of emotions is evidenced by the fact that they receive prioritized attention at early stages of processing the environment in humans and some other primates. Nevertheless, we do not fully understand how emotion modulates attention over longer durations in primates, particularly in great apes. Bonobos, one of our closest relatives, stand out in emotion processing and regulation among great apes. This makes them an interesting comparison species and a valuable model for studying the evolution of emotion perception in hominids. We investigated how bonobos and humans spontaneously attend to emotionally valent scenes in a preferential looking task using eye-tracking. With Bayesian mixed modelling, we found that bonobos and humans generally looked longer at emotional scenes, mainly of conspecifics. Moreover, while bonobos did not have a bias toward emotional human scenes, humans sustained their attention toward bonobos playing, grooming and having sex. Furthermore, when exploring an immediate bias for emotions, humans showed a bias toward affiliative human scenes, and bonobos showed a bias away from bonobos-in-distress scenes. These findings suggest that emotions modulate attention at early and later attentional stages in bonobos, similar to humans.

## 1. Introduction

Emotional expressions are the conduit through which information about the expressor’s internal state and motivations are communicated to others. Perceiving emotions is therefore an adaptive process that is crucial to humans and other social animals [1]. To date, no single definition of emotion is universally accepted. Nevertheless, broadly defined, emotions are adaptive states that are caused by external, biologically relevant stimuli that trigger a repertoire of physiological, behavioural and cognitive changes in an individual [2]. In humans, emotions are also linked with subjective experiences (feelings), which may or may not be present in other animals as well [2]. Despite the lack of a clear definition, all mammals likely share homologous emotional brain networks [3], and over a century ago, Charles Darwin theorized that emotional expressions are universally shared among certain animals [4]. As such, it is likely that the mechanisms underlying emotional processes are shared between closely related species as well.

On a cognitive level, emotions drive several mechanisms like memory, learning, attention and decision-making [5]. In humans, emotional information is so important that the brain prioritizes its processing even when attentional resources are limited [6,7]. For instance, emotionally salient information such as smiles or angry faces is preferentially remembered and immediately attracts attention [8]. This selective attention for emotional signals also extends to whole body emotional expressions [9] and to emotional scenes [10]. There is now also a growing body of evidence that emotion-biased attention is present in other primates, including our closest living relatives—the great apes [11–14]. For instance, rhesus macaques (*Macaca mulatta*) immediately attend to threatening facial expressions [15,16], and bonobos (*Pan paniscus*) to affiliative scenes [11]. Similarly, emotionally valent stimuli such as play faces impact bonobos’ performance on an emotional Stroop task [17], and a similar effect has been found for chimpanzees (*Pan troglodytes*), gorillas (*Gorilla gorilla*) and Japanese macaques (*Macaca fuscata*) when presented with images of snakes or veterinarians [18,19]. These findings suggest that emotion-biased attention is an evolutionarily old mechanism that is shared at least within some primate species [6].

The findings are promising, but the mechanisms of selective attention for emotions in great apes require further research owing to gaps in our knowledge and inconsistencies in the results. For example, some studies employ a dot-probe task to measure how emotions modulate attention [20]. In the task, participants are briefly shown two simultaneously presented stimuli (e.g. for 300 ms), typically an emotional and neutral one. A ‘dot-probe’ then replaces one of the two stimuli, and response times on pressing the dot-probe reflect the initial location of attention. Two studies found that bonobos attend faster to emotionally valent scenes of others compared with neutral scenes [11], and especially of unfamiliar conspecifics [12]. However, this effect has not been found in chimpanzees [21,22], nor in orangutans (*Pongo pygmaeus*) [23] using the same task. Additionally, one study found an attention bias to unfamiliar human faces in chimpanzees and gorillas, but this effect disappeared when the faces showed a surprised expression [24]. Nevertheless, in tasks where stimuli are presented for longer durations (e.g. looking time tasks), chimpanzees gaze longer at negatively valenced stimuli such as agonistic interactions [25]. Similarly, one study showed that orangutans and human children looked longer at fearful human expressions, and the silent bared-teeth display of orangutans [26]. To date, there are no studies investigating looking time toward emotional stimuli of conspecifics or humans in bonobos, nor in gorillas. Moreover, the used tasks likely tap into different attentional mechanisms. Dot-probe tasks provide a snapshot of where attention was allocated first, whereas tasks that present stimuli for longer durations can measure the maintenance of attention toward these stimuli [27]. It is therefore premature to draw definitive conclusions on how emotion modulates attention in great apes, but eye-tracking could be a suitable method to bridge some of the divergent findings as it enables the tracking of attention over time.

Indeed, advances in the development of non-invasive eye-tracking for primates have already led to fruitful results within the domain of social cognition [28], and to methodological recommendations for research with primates [29]. Currently, there is almost no work looking into how emotions may modulate attention over time, which is therefore the aim of the current project. Using a comparative framework, we examine emotion-biased attention in humans and one of our closest relatives, bonobos. Like humans, bonobos have well-developed brain areas involved in social cognition, with a high degree of connectivity and volume in the amygdala (regulating emotions, attention, memory and social decision-making) and subgenual anterior cingulate cortex (regulating positive affect and arousal) [30]. Additionally, bonobos have a high social sensitivity and perform well on tasks in which they have to take others’ perspectives, indicating they may be attuned to the emotional needs of others [31]. Moreover, it is the only great ape in which an immediate attention bias was found toward emotional expressions of conspecifics [11,12]. All these characteristics make bonobos an interesting species for comparisons with humans, as these comparisons may help us improve our understanding of how emotions modulate cognitive mechanisms such as attention across phylogenetically close species. Moreover, by making comparisons between related species, we can gain more knowledge on the evolution of emotion perception in hominids.

How do emotional expressions of bonobos and humans compare? While there are some evolutionary continuities in expressions (e.g. the relaxed open-mouth play face for affiliative interactions, a nose wrinkle when disgusted), there are also marked differences in when and why these expressions occur [32]. For instance, the vast literature on emotional expressions in humans show that similar expressions can have different meanings across context (e.g. smiling is an affiliative expression, but can also indicate subordination or embarrassment [33]). Similarly, a bared-teeth display in bonobos and chimpanzees can signal benign intentions, and is expressed in both affiliative situations (e.g. during play) and agonistic situations [34]. To make interspecies comparisons possible, we therefore chose to consolidate facial and bodily expressions into categories that likely convey a similar emotional state in our study. Specifically, we looked at how bonobos and humans view scenes depicting individuals in distress (e.g. crying in humans and bared-teeth displays in bonobos), affiliative scenes such as grooming (bonobos) and embracing (humans), arousing scenes showing individuals engaged in sexual activities or showing aroused genitalia (bonobos), individuals kissing and involved in a romantic embrace (humans), and finally, individuals yawning. We included yawning as it is highly contagious in bonobos and humans [35–37], and its contagiousness is linked to social closeness, therefore potentially serving a social function [38]. Furthermore, yawns capture immediate attention in bonobos [11].

Using these types of socio-emotional scenes, we here set out to investigate whether bonobos and humans attend longer to emotional scenes when they are presented alongside neutral scenes. Moreover, we presented both species with emotional and neutral scenes of conspecifics and heterospecifics (i.e. the other species). Prior results in humans showed an immediate attention bias toward bonobo emotional scenes [10]. We do not yet know whether bonobos have a similar bias toward human emotional scenes, but it is a possibility given their high social sensitivity [31]. We therefore predicted that bonobos and humans would attend longer to emotional scenes compared with neutral scenes irrespective of the displayed species. We also expected this effect to persist for the separate species, but expected it to be more pronounced for conspecifics’ scenes, as emotional expressions are typically used within the context of one’s own species [32]. Furthermore, we explored potential differences between the different emotion categories, as previous results showed an immediate attention bias toward positive scenes in bonobos [11], and positive and negative expressions in humans [39]. After peer-review feedback, we additionally explored whether both species initially orient their attention toward emotional scenes of conspecifics and heterospecifics as well.

## 2. Methods

### (a) Participants

#### (i) Bonobos

Four bonobos (all female, 12–28 years, *M* = 16.25, s.d. = 9.03, electronic supplementary material, table S1) that are part of a social group of 12 individuals housed in the primate park Apenheul, Apeldoorn, The Netherlands, participated. Three bonobos had prior experience with taking part in touchscreen-based research [11,12]. All bonobos were new to eye-tracking (see electronic supplementary material for more information on subjects and housing).

#### (ii) Humans

Participants were primarily Dutch visitors of primate park Apenheul and were recruited via opportunistic sampling. In total, 99 adults participated (age category 18–20: *n* = 9, 21–30: *n* = 48, 31–40: *n* = 9, 41–50: *n* = 24, 51–60: *n* = 7, 61–70: *n* = 1, 71–80: *n* = 1; 58 women, 41 men; see electronic supplementary material for more information on recruitment and test environment).

### (b) Stimuli

Bonobo and human participants were presented with the same stimulus set consisting of bonobo and human scenes, paired as bonobo-emotional and bonobo-neutral scenes and human-emotional and human-neutral scenes. Stimuli were colour pictures selected from previously validated sets, sampled from the internet (bonobos: [11], humans: [10]). Three bonobos had seen the bonobo scenes 2 years prior in a dot-probe task, but not the human scenes. Human participants did not know the depicted individuals (bonobo nor human). Stimuli had a dimension of 500 × 430 pixels (or 430 × 500 pixels), and were matched on number of individuals visible, distance from the camera, full body versus face only, type of background (e.g. grass versus concrete), dimension (horizontal versus vertical) and luminance (see electronic supplementary material, tables S3 and S4 for an overview of the number of depicted individuals, age and sex). For each stimulus pair, we created a black background upon which the stimuli were presented on either side of the screen.

#### (i) Bonobo scenes

Although it can be argued that we do not exactly know what bonobo emotions are, we do know the social relevance of certain facial expressions (e.g. the fear-grin, relaxed open-mouth play face and yawning [40], and socio-emotional behaviours (sex, grooming) [41]). Therefore, emotional scenes consisted of bonobos playing, or bonobos having sex or displaying an erection (male) or a large swelling (female) (all included in the sex category), displaying distress, grooming and yawning (see electronic supplementary material for an elaboration on why we included these categories). Neutral scenes were social, but emotionally neutral and consisted of bonobos lying down, sitting or walking with a neutral facial expression (electronic supplementary material, figure S1 and table S2). In total, there were 10 unique bonobo scenes for the emotional categories distress, grooming, sex, 9 for play and 11 for yawning, resulting in 50 unique emotional scenes that we subsequently paired with 50 unique neutral scenes, totalling to 50 unique stimulus pairs depicting only bonobos. All scenes were rated on valence and arousal by a group of bonobo experts in a previous study [11] (electronic supplementary material, table S5).

#### (ii) Human scenes

To make comparisons between bonobos and humans possible, we selected colour pictures of human emotional scenes that were an approximation of the emotional bonobo scenes, conveying a similar emotional state. We included *play, erotic, distress, embracing* and *yawning* scenes (electronic supplementary material, figure S2). There is no human equivalent of grooming, so we opted to use embracing as it reflects affection and social closeness and involves physical contact, just like grooming [42]. Moreover, the erotic scenes depicted two individuals kissing while lying in bed, partially undressed. Neutral scenes depicted individuals lying down on grass, sitting, walking or cycling with a neutral facial expression (see electronic supplementary material, tables S2 and S4). All scenes were non-acted, and rated on valence and arousal by other participants in a previous study (electronic supplementary material, table S5) [10]. Like the bonobo-only scenes, we had 50 unique stimulus pairs depicting only humans, thus 100 unique stimulus pairs in total (bonobo-only and human-only combined).

### (c) Procedure

#### (i) Bonobos

Testing took place in the bonobos’ night enclosure where we installed a wooden box that housed a monitor, a webcam to film the bonobos, the eye-tracker and the juice tube to keep the bonobos’ head relatively still during testing (electronic supplementary material, figure S3). Testing started by calling forth a bonobo by their name (see electronic supplementary material for more details).

Bonobos were calibrated and tested using Tobii Studio (v. 3.4.8) using a 2-point calibration with a Tobii X2−60 eye tracker and a 4:3 (1280 × 1024 pixels) monitor. This calibration is commonly used in animal research, and is deemed sufficient for tracking gaze in Tobii systems [29]. Calibrations were accepted when the software indicated that the data were closely centred around the calibration points [29], and the first successful calibration for each individuals was used for all subsequent tests (see electronic supplementary material, figures S4–S6).

The trial procedure was semi-automated and started with a 9-point grid to check for calibration accuracy, shown until the experimenter manually continued the experiment (electronic supplementary material, figure S3). The grid presented things that were familiar and attractive to the bonobos (e.g. a turtle, an infant bonobo head, a grape and a piece of pineapple) and served to attract attention. A black screen was subsequently displayed for 4 s, automatically followed by a fixation video (a sped-up nature movie) positioned in the middle of the screen. Only when the participant’s fixation was on the video for more than 1 s, the experimenter manually continued the trial, leading to the presentation of the stimuli. This was done to ensure attention to the middle of the screen right before stimulus onset. An emotional and neutral scene were then simultaneously presented for 3 s on each side of the screen, in accordance with previous eye-tracking tasks with non-human great apes [29]. Hereafter, a black screen was automatically shown (4 s), and this concluded a trial. After 10 trials, the task ended automatically. Bonobos were continuously provided with small squirts of diluted juice during testing, which happened independent of their viewing performance. Furthermore, they first completed all trials with bonobo scenes before moving on to trials with human scenes.

Our stimulus set consisted of 100 unique emotional–neutral stimulus pairs depicting bonobos (50 pairs) or humans (50 pairs). This resulted in 100 unique trials divided over sessions of 10 trials each. Each bonobo completed a maximum of two sessions per day. The order of trials was randomized by the experimenter beforehand using a random number generator. The location of the two scenes on the left or right side of the screen was counterbalanced. Each bonobo received the same randomized order of trials, and initially completed all 50 trials depicting only bonobos. To pre-emptively compensate for data loss, we repeated each trial on average 4.5 times for each bonobo. After completing all bonobo-only trials (including repetitions), the bonobos completed all 50 trials depicting only humans, and similarly, these trials were repeated 3.46 times on average (see electronic supplementary material, table S6). On average, each bonobo completed 397.75 trials (s.d. = 37.30, electronic supplementary material, table S6), and 1591 trials in total.

#### (ii) Humans

Participants provided written consent to participate in the study, and were tested using a 19’ laptop (1920 × 1200 pixels) and a Tobii X2−60 eye tracker with Tobii Studio. The set-up was placed inside a small booth on a table in an indoor location (see electronic supplementary material for more details). The eye tracker was calibrated using the 5-point automated calibration procedure in Tobii Studio. Calibrations were accepted when the output showed that the data were closely centred around the calibration points [29]. To keep testing time to a minimum (zoo visitors were voluntarily taking part in our study), participants completed 30 trials in one session, and the presentation of bonobo and human scenes was counterbalanced within each of the 10 versions of the task and the emotional categories randomized across trials (see electronic supplementary material). Moreover, because the bonobos could not be instructed, human participants also received minimal instructions. Participants were informed that they should pay attention to the screen, keep looking at the fixation video once it appeared, and not move their head too much. The trial procedure differed from the bonobos in that the sequence was fully automated. The fixation video was shown for a fixed time (3 s), and participants could take a short break between every set of 10 trials where they were allowed to move their head, but were requested to remain seated. At the end of the last set of 10 trials, participants saw a screen on which they were thanked for their participation. They were subsequently debriefed about the study.

### (d) Data preparation

#### (i) Bonobos’ data

To assess calibration accuracy over time, we checked whether the raw fixation data per bonobo and per session reasonably matched with the areas of the stimuli on the screen and found that for two bonobos, in some sessions, there were consistent shifts in gaze data to the left or to the right relative to the position of the stimuli on the screen. We used k-means clustering to establish the specific offsets, and corrected 37/54 sessions for Monyama (average offset of +134 pixels), and 39/46 sessions for Zuani (average offset of −141 pixels) (see electronic supplementary material for more details). Next, two square regions of interest (ROIs) were defined, covering each stimulus (electronic supplementary material, figure S6). We then extracted the *Total Fixation Duration* (TFD) and *Time To First Fixation* (TTFF) per ROI using the Tobii Fixation Filter. The TFD indicates how long the bonobos looked at each ROI, whereas the TTFF reflects the time until the first fixation after stimulus onset falls on one of the ROIs.

For our TFD measure, 155 trials were removed because no gaze data were collected. An additional 16 trials were removed where the total fixation duration was higher than the stimulus presentation of 3 s (*M* = 4.60 s, s.d. = 1.11), likely owing to Tobii registering a fixation that extended beyond the maximum stimulus duration. This resulted in 1420 valid trials (and removal of 10.7% of the data).

For our TTFF measure, we additionally checked whether the final fixations before stimulus onset fell in the middle of the screen (i.e. on our fixation video). We added this extra check because there were instances where the bonobos managed to look away from the screen in the short time period between looking in the middle of the screen and directly after stimulus onset. These instances would result in their fixations not falling in the middle of the screen during the last 500 ms of video presentation and/or extremely short (<80 ms) [43] TTFF values for our ROIs. Out of the original 1591 trials, we removed 681 (42.8%) for this reason. Furthermore, an additional 155 trials were removed because no data were collected during stimulus presentation (see electronic supplementary material for more details). In total, we removed 836 trials (52.5%), leaving a total of 755 valid trials. While significant compared with human studies or those conducted in research institutes, this is mainly due to the context in which we test; the bonobos were tested in their natural group with minimal interventions, increasing the chance of disruption, but also keeping anxiety to a minimum.

#### (ii) Humans’ data

Similar to the bonobos, we created ROIs in Tobii Studio, and extracted data on TFD and TTFF per ROI using the Tobii Fixation Filter. After data collection finished, we realized that in versions 3, 6 and 9 of the task, we accidentally showed one stimulus twice. These repetitions were removed from further analyses (31 trials out of 3002; 1.03%). Furthermore, for five participants, there was a technical malfunction with the eye tracker resulting in 60% or more data loss. These participants were excluded from further analyses (150 trials, 4.99%). For our TFD measure, we removed an additional trial (0.03%) because the TFD exceeded 3 s, and an additional 40 trials in which no data were collected (1.33%). This resulted in 2780 valid trials (92.6%) for analysis. For our TTFF measure, we removed trials in which the TTFF was shorter than 80 ms (1178 trials, 39%), meaning that participant’s attention was already at the location of one of the stimuli at stimulus onset. This left a total of 1643 valid trials (54.6%) for analysis.

### (e) Statistical analyses

We used a Bayesian mixed modelling approach in R Statistics v. 4.2.2 and using the brms package [44]. We were interested in examining an immediate attention bias and a sustained attention bias toward emotional scenes depicting either bonobos or humans, in bonobo and human participants. We examined the data of bonobo and human participants separately, as bonobos received multiple sessions per testing day and a large overall number of trials, whereas humans only completed 30 trials in one session. For both species, we examined whether: (i) there was an overall and initial bias toward emotions, (ii) a bias specifically for emotional bonobo scenes and/or emotional human scenes compared with neutral scenes of the same species, (iii) whether the bias toward emotional scenes was stronger for one or the other species and (iv) a bias for specific emotion categories within the bonobo and human scenes. Finally, in each analysis, we controlled for the location of the emotional scene on the screen (left or right), as previous research suggests that humans and some non-human animals may have a left-sided visuospatial bias [45]. The results for side biases are reported in the electronic supplementary material.

We used weakly informative Gaussian priors for all our models, specifically a Student’s *t* prior (default) (*d.f.* = 3, *M* = 0, s.d. = 2.5) for the standard deviation coefficient, and a normal distribution (*M* = 0, s.d. = 1) for all other coefficients. All independent variables were sum coded, allowing us to compare the mean of one group with the grand mean of all groups.

Furthermore, we report the median estimate coefficient (Mdn), together with the 89% credible interval (CrI), which is the narrowest interval that contains 89% of the posterior probability density function. Therefore, the CrI is the range of values that we are 89% certain contains the true value of the estimated parameter, based on our data. This is different from the frequentist confidence interval, which quantifies our belief that if we were to repeat the experiment many times, a certain percentage (e.g. 95%) of intervals would contain the true value of the parameter. We also report the probability of direction (pd), which indicates the certainty that an effect goes in a specific direction [46]. Additionally, to establish model convergence, we followed the Bayesian statistics guidelines set out by Depaoli and Van de Schoot [47]. We assessed trace and autocorrelation plots, the Gelman–Rubin diagnostic values (convergence indicated by a value close to 1) and density histograms for the posterior distributions. Finally, to sample the posterior distribution we used 10 k iterations including 2 k warmups.

#### (i) Total fixation duration in bonobos

To quantify sustained attention toward emotional scenes in bonobos, we calculated the *proportional looking duration for emotional scenes* (from here on: PLD_emotion_) using the following formula:


$$\frac{looking\;duration\;for\;emotional\;scene}{looking\;duration\;for\;emotional\;scene+looking\;duration\;for\;neutral\;scene}$$

Based on this formula, PLD_emotion_ could vary between 0 and 1; a PLD_emotion_ higher than 0.5 would indicate a longer looking duration for the emotional scene.

The bonobos did not always continuously attend to the screen, and disruptions by group members occurred as they were tested in their natural group setting. To strike a balance between data preservation and including only valid data, we calculated a weight for each trial. We calculated the weight according to the following formula:


$$\frac{looking\;duration\;for\;emotional+neutral\;scene\;within\;a\;trial}{average\;looking\;duration\;for\;emotional+neutral\;scene\;per\;subject}$$

The weight gives more importance to trials in which participants paid more attention to the scenes, and less importance to trials where participants were relatively inattentive or where data were missing. Within a 3 s trial, bonobos on average looked 1.93 (s.d. = 0.78) s at the bonobo scenes, and 2.04 s (s.d. = 0.72) at the human scenes.

We used zero-one-inflated beta (ZOIB) regression to account for 0s, 1s and the data between the range [0 , 1]. For our measure of interest—proportional looking duration for emotional stimuli (PLD_emotion_) across trials—we ran a model including the location of the emotional scene on the screen (left or right), and an interaction between *species on the scene* (bonobo or human) and the *emotional category* (distress, groom, play, sex/erotic or yawn). We included *session* nested within *subject* as a random effect, but owing to computational issues, we did not include a random slope for species nor for emotional category. Furthermore, we included the independent variables precision (*phi*), zero-or-one inflation probability (*zoi*) and conditional-one probability (*coi*) [48] as part of the ZOIB regression.

#### (ii) Total fixation duration in humans

The analysis procedure for humans was similar to that for the bonobos; we first calculated the PLD_emotion_. Within the 3 s trial window, human participants looked on average 2.66 s (s.d. = 0.38) at the human scenes, and 2.64 s (s.d. = 0.43) at the bonobo scenes. Similar to the bonobos, we calculated the weight of a trial depending on how long a participant looked at the scenes relative to their average looking duration for all the scenes (*M* = 1, s.d. = 0.15, range (0.01–1.51)). Finally, we also included the independent variables for *phi, zoi* and *coi* [48].

#### (iii) First fixation location in bonobos

To quantify an immediate attention bias for emotional scenes, we examined the first fixation location of the bonobos based on the TTFF value we acquired from Tobii Studio. We transformed these values into a binary variable: if the TTFF for an emotional scene was shorter than for a neutral scene, we scored this trials as a first fixation falling on the emotional scene and *vice versa*. Our first dependent variable was therefore *first fixation location* (1 = *emotion*, 0 = *neutral*). Logistic regression was used to analyse the relationship between our dependent variable and the following independent variables: the *location of the emotional scene* on the screen (left or right), and an interaction between the *species in the scene* (human or bonobo), and the *emotion category* (distress, grooming/embracing, play, sex/erotic or yawn). Furthermore, we included *cumulative session* nested in *subject* as a random effect, and included random slopes for *emotion category* and *species*.

#### (iv) First fixation location in humans

For humans, we had the same approach as for bonobos, with TTFF as our dependent variable. We ran a similar model with *location of the emotional scene* (left or right), and an interaction between *species in the scene* (human or bonobo) and the *emotion category* (distress, grooming/embracing, play, sex/erotic or yawn). We included *subject* as a random effect, and similar to the bonobos, random slopes for *emotion category* and *species*.

## 3. Results

### (a) Sustained attention bias

#### (i) Bonobos

**Figure 1.**
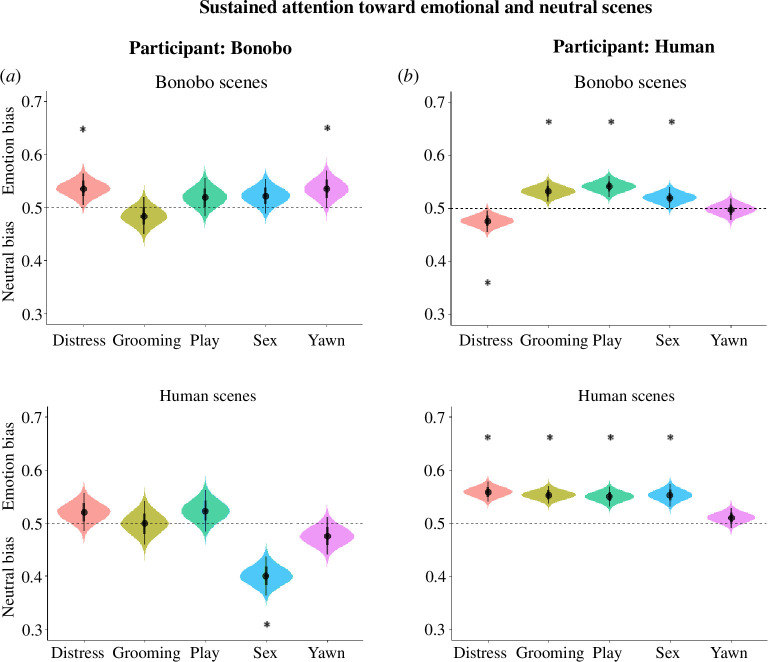
Violin plot displaying a sustained attention bias toward emotional or neutral scenes in bonobos (*a*) and humans (*b*). The curves’ widths depict the data’s distribution (89% *CrI*). Dots represent the median PDL_emotion_. Curves above 0.50 indicate looking longer at emotional scenes; below 0.50 looking away from emotional scenes/toward neutral scenes. Asterisks indicate robust effects.

Overall, bonobos did not reliably look longer at emotional compared with neutral scenes (*Mdn* = 0.501, *89% CrI* (0.492–0.511), *pd* = 0.59, electronic supplementary material, tables S11 and S12, and figure 1). However, they did reliably look longer at emotional bonobo scenes compared with neutral scenes (*Mdn* = 0.519, *89% CrI* (0.507–0.531), *pd* = 0.99). Conversely, bonobos looked longer at neutral human scenes when compared with emotional scenes (*Mdn* = 0.484, *89% CrI* (0.469–0.498), *pd* = 0.97). Nevertheless, both effects are barely within the boundaries of what is considered reliable, as the credible interval is near 0.50. Furthermore, contrasting the looking duration for emotional scenes of bonobos and humans, bonobos looked 3.5 percentage points longer at emotional scenes of bonobos compared with human scenes (*Mdn*_bonobo–human_ = 0.035 , *89% HDI* (0.016–0.054), *pd* = 1.00).

When examining specific emotion categories, bonobos reliably looked longer at distressed bonobos compared with neutral stimuli (*Mdn* = 0.535, *89% CrI* (0.511–0.559), *pd* = 0.99, electronic supplementary material, tables S11 and S12, and figure 1). Moreover, bonobos reliably looked longer at bonobo yawn scenes compared with neutral scenes (*Mdn* = 0.535, *89% CrI* (0.506–0.563), *pd* = 0.97). Finally, bonobos reliably looked longer at neutral stimuli when they were paired with erotic human scenes (*Mdn* = 0.400, *89% CrI* (0.371–0.430), *pd* = 1.00).

#### (ii) Humans

Human participants reliably looked longer at emotional scenes (*Mdn* = 0.529, *89% CrI* (0.524–0.535), *pd* = 1.00, electronic supplementary material, tables S13 and S14, and figure 1). Furthermore, this was true for both human (*Mdn* = 0.545, *89% CrI* (0.538–0.552), *pd* = 1.00) and bonobo scenes (*Mdn* = 0.514, *89% CrI* (0.506–0.552), *pd* = 0.98). Contrasting human and bonobo scenes, participants looked 3.2 percentage points longer at emotional human scenes compared with emotional bonobo scenes (*Mdn*_human–bonobo_ = 0.032, *89% HDI* (0.021–0.042), *pd* = 1.00). In short, participants overall looked longer at emotional scenes, but slightly longer at emotional human compared with bonobo scenes.

When examining specific emotion categories and depicted species, participants were more likely to look at distress, embracing, play and erotic scenes of humans compared with neutral scenes (distress: *Mdn* = 0.559, *89% CrI* (0.544–0.574), *pd* = 1.00; embracing: *Mdn* = 0.553, *89% CrI* (0.540–0.567), *pd* = 1.00; play: *Mdn* = 0.550, *89% CrI* (0.535–0.565), *pd* = 1.00; erotic: *Mdn* = 0.553, *89% CrI* (0.536–0.570), *pd* = 1.00), but not to yawn scenes (*Mdn* = 0.551, *89% CrI* (0.495–0.526), *pd* = 0.86; electronic supplementary material, tables S13 and S14, and figure 1). Participants also looked longer at grooming (*Mdn* = 0.532, *89% CrI* (0.516–0.548), *pd* = 1.00), play (*Mdn* = 0.541, *89% CrI* (0.525–0.558), *pd* = 1.00) and sex scenes (*Mdn* = 0.520, *89% CrI* (0.504–0.536), *pd* = 0.98) of bonobos. Furthermore, participants looked longer at neutral bonobo scenes compared with distress scenes (*Mdn* = 0.476, *89% CrI* (0.460–0.493), *pd* = 0.99). Finally, participants did not look longer at bonobo yawns compared with neutral scenes (*Mdn* = 0.498, *89% CrI* (0.481–0.515), *pd* = 0.57).

### (b) Immediate attention bias

#### (i) Bonobos

**Figure 2.**
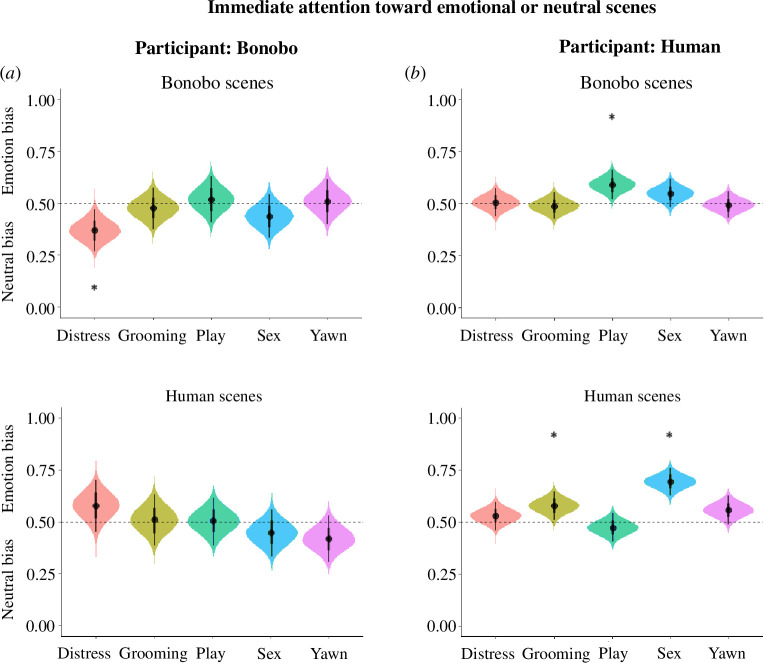
Violin plot displaying an immediate attention bias toward emotional or neutral scenes in bonobos (*a*) and humans (*b*). The curves’ widths depict the data’s distribution (*89% CrI*). Dots represent the median first fixation bias toward emotional or neutral scenes. Curves above 0.50 indicate an immediate attention bias toward emotional scenes; below 0.50 a bias toward neutral scenes. Asterisks indicate robust effects.

Bonobos did not show an overall initial attention bias toward emotional scenes compared with neutral scenes (*Mdn* = 0.476, *89% CrI* (0.446–0.504), *pd* = 0.908, electronic supplementary material, tables S7 and S8, and figure 2). When specifically comparing an initial attention bias toward emotional bonobo scenes with neutral scenes, we found that bonobos initially tended to fixate on neutral scenes first (*Mdn* = 0.461, *89% CrI* (0.423–0.500), *pd* = 0.946). However, the effect was not very robust as the upper boundary of the credible interval includes 0.50. Furthermore, bonobos did not have an initial bias for emotional human scenes compared with neutral scenes (*Mdn* = 0.490, *89% CrI* (0.446–0.634), *pd* = 0.557). Next, when contrasting the likelihood of the first fixation falling on emotional bonobo scenes to the likelihood of the first fixation falling on emotional human scenes, we did not find a robust difference (*Mdn_bonobo-human_* = −0.029, *89% HDI* (-0.088–0.029), *pd* = 0.79). When examining the emotion categories and the depicted species, the first fixation of bonobos was more likely to fall on a neutral bonobo scene compared with the bonobo *distress* scene (*Mdn* = 0.369, *89% CrI* (0.287–0.450), *pd* = 0.993, electronic supplementary material, tables S7 and S8, and figure 2). We did not find an effect for the other categories, nor for the human scenes. Therefore, bonobos seemed to initially fixate *away* from distressed bonobo scenes.

#### (ii) Humans

Participants initially fixated more on emotional scenes than neutral stimuli (*Mdn* = 0.55, *89% CrI* (0.527–0.562), *pd* = 1.00, electronic supplementary material, tables S9 and S10 and figure 2). When distinguishing between depicted species, participants were more likely to initially fixate on emotional human scenes compared with neutral scenes (*Mdn* = 0.57, *89% CrI* (0.541–0.591), *pd* = 1.00). For bonobo scenes, there was no clear bias in initial first fixation bias toward emotional scenes (*Mdn* = 0.52, *89% CrI* (0.499–0.548), *pd* = 0.94). This result was further corroborated with the finding that participants showed more initial first fixations on emotional human scenes than on emotional bonobo scenes (*Mdn_human-bonobo_* = 0.042, 89% *HDI* (0.007–0.077), *pd* = 0.97). In other words, our human participants appeared to have an initial fixation bias toward emotional scenes, but mainly when they depicted humans.

Examining the emotional categories, participants initially fixated on human embracing and erotic scenes compared with neutral scenes (embracing: *Mdn* = 0.58, *89% CrI* (0.521–0.634), *pd* = 0.98, erotic: *Mdn* = 0.69, *89% CrI* (0.693–0.746), *pd* = 1.00, electronic supplementary material, tables S9 and S10, and figure 2). Furthermore, participants initially fixated more on bonobo play scenes compared with neutral scenes (*Mdn* = 0.59, 89% *CrI* (0.532–0.647), *pd* = 0.99). In short, the participants had an immediate attention bias toward human embracing and erotic scenes, and bonobo play scenes.

## 4. Discussion

Emotions and their perception in non-human animals are intriguing, yet elusive [2]. Here, we examined sustained attention for emotional scenes in bonobos and humans. After reviewer feedback, we also explored immediate attention for emotional scenes. Initially, the bonobos tended to shift attention away from bonobo distress scenes, whereas humans displayed an initial bias toward emotional scenes, mainly of humans. Hereafter, bonobos and humans kept looking at (some) emotional scenes, predominantly of conspecifics. Below, we discuss our results on immediate attention for emotions, followed by the results on sustained attention. Moreover, we first discuss the bonobo findings, followed by the human findings.

### (a) Immediate attention

Although bonobos did not show an immediate bias toward emotional scenes in general, the bias *away* from distressed bonobo scenes was strong. This finding is surprising, as we found an initial bias toward positively valenced emotions in a previous study [11]. However, this discrepancy can likely be explained by methodological differences. Firstly, the previous study employed a dot-probe task in which reaction time on a dot-probe is a proxy for the initial location of attention [49]. Importantly, the reaction time on dots replacing emotional and neutral bonobo scenes were compared across trials rather than within-trial (which is what we did), and bonobo scenes were paired with control scenes of other animals. We directly tested how emotional and social, but emotionally neutral scenes competed for attention, which makes comparing results difficult at this stage. Although challenging, it could be interesting to combine eye-tracking measures with reaction time paradigms in non-human great apes to see how the two measures are related.

Why did the bonobos initially orient away from distress? Possibly, these scenes were initially very intense to the bonobos, which is plausible given that the experts rated these stimuli as more intense than other emotional categories (electronic supplementary material, table S5). Nevertheless, this means the bonobos must have detected the scenes in the first place, but it is possible to rapidly detect information in the periphery via covert attention [50]. We currently do not know to what extent this is true for non-human great apes, but this could be an interesting avenue for future work.

The bonobos did not have an immediate or sustained bias to any of the human emotional scenes. Yet, they also only showed an initial bias away from distressed bonobos, and not for the other categories. Possibly, the human emotional scenes were not salient enough to trigger rapid attention allocation [32]. This is one of the first studies to investigate an immediate attention bias for human emotional expressions in great apes (though see [24]), but our work aligns with earlier findings. In a dot-probe task using familiar and unfamiliar human faces expressing six basic emotions (anger, fear, happiness, sadness, surprise and disgust), bonobos also did not show a bias toward these expressions (Zijlstra, T. W., Van Berlo, E., Roth, T. S., & Kret, M. E., 2024, unpublished results). Nevertheless, it is too early to draw conclusions. One recent study with chimpanzees and gorillas looked into attention biases for familiar and unfamiliar human faces with a neutral expression or a surprised expression. Findings showed a bias toward unfamiliar neutral faces, but this effect disappeared for unfamiliar faces with a surprised expression [24]. Although the emotion did not cause an immediate bias away or toward it, it did appear to modulate the bias toward unfamiliar faces. Whether an immediate attention bias toward emotions in non-human great apes is restricted to conspecifics therefore remains an open question. More research is needed on cross-species emotion recognition, and specifically, the modulating effects of different variant and invariant facial characteristics (for instance, familiarity: [12,24]).

Human participants showed an immediate attention bias toward emotions, mainly of conspecifics, and particularly to positive scenes (erotic and embracing), in line with previous work [10]. These findings suggest that attention is not exclusively biased toward threatening or negative stimuli. Instead, positive stimuli can bias attention because they elicit arousal and because they are biologically or motivationally relevant to an individual [51]. Indeed, a meta-analysis indicated that erotic pictures and pictures of babies, money and favoured food elicit a stronger attention bias than smiles or generally positive stimuli [39]. In our study, erotic stimuli elicited the strongest immediate attention bias, similar to a dot-probe study using the same stimulus set [10]. Moreover, consistent with the literature on healthy human adults [52], we did not find an immediate bias toward or away from distress scenes in humans, even though bonobos clearly showed a bias away from distress. In reaction time studies on healthy humans, evidence for a bias toward or away from negative expressions is often absent, but perceived level of threat can modulate it in anxious individuals [53]. While we used previously validated stimuli (see [10]), linking individual ratings to individual performances could provide insights into individual emotional biases in future studies.

Human participants also showed an immediate attention bias toward playful bonobo scenes; a positively valenced scene and scored as such in our previous study [10]. The play face of humans and bonobos share morphological similarities [32] and potentially elicited the bias, although a similar bias would then be expected for human play scenes, which did not occur. In our previous dot-probe study, we also did not find an immediate bias toward play scenes [10], but there, bonobo play scenes scored higher on the arousal scale than other categories, and higher than human play scenes. The perceived arousal of stimuli can greatly influence immediate attention biases in humans [39], which may explain why our participants had an immediate bias toward playful bonobo scenes.

Overall, these findings suggest that in humans attentional priority is given to emotional signals, which may extend to positive emotional signals of bonobos. Possibly, the continuity between expressions in humans and other great apes activates similar attentional mechanisms that help with adequately attending to conspecifics’ expressions. In line with this idea, a recent study found that human empathy towards other animals is linked with phylogenetic closeness, mainly because of an overlap in ‘human-like’ traits [54]. Nevertheless, little is known about the evolution of our sensitivity toward emotional signals from other beings, so this topic requires more exploration.

### (b) Sustained attention

Overall, bonobos looked longer at emotional versus neutral scenes. Specifically, they looked longest at potentially negative scenes of conspecifics yawning or in distress. Yawning can indeed signal distress [55], possibly communicate dominance in some primates [56], but also occur during resting [57] and is highly contagious [58]. Furthermore, yawns bias attention in bonobos and humans [10,11]. Moreover, human infants can already distinguish yawns from other mouth movements [59], suggesting that detecting yawns has some biological significance [57]. There are several potential explanations for our findings. First, one might argue that still images of yawns are similar to still images of aggressive faces, and therefore hold attention. All bonobo yawning stimuli displayed an open mouth with the teeth visible, similar to expressions of aggression. However, unlike the intense stare of aggressive faces, yawns involve more relaxed, squinted or shut eyes [32]. These are subtle, yet crucial differences, but it remains to be investigated whether bonobos can detect these differences from still images. Second, yawning may serve as a cue, therefore holding attention. Yawns regulate brain temperature and may induce vigilance in the yawner through this thermoregulatory function [57]. Detecting others’ yawns may subsequently enhance the vigilance of observes via contagious yawning [57], and therefore potentially bias attention.

The bonobos sustained their attention toward distress scenes; this finding aligns well with current literature on their emotional capacities. For instance, bonobos are sensitive to the emotional needs of others and console them when in distress [60]. Moreover, bonobos have a high social tolerance and cooperate with strangers [61], suggesting they could be sensitive to the emotional needs of strangers as well. Consolation between unaffiliated individuals in the wild has not yet been observed, but our results at least indicate that bonobos may be attentive to strangers in distress, although we do not yet know whether this is because of perceived threat or because of the bonobos’ other-regarding tendencies (or both).

We expected a similar but less pronounced attentional bias pattern when the bonobos viewed human emotional scenes. Despite lacking strong evidence for bonobos’ prolonged gaze at human emotions, their viewing patterns for human distress, embracing and play scenes were similar to those for the bonobo equivalents. Possibly, this is due to the similar morphological action tendencies of human and bonobo expressions of distress and playfulness. For instance, a general feature of fearful expressions in humans and other primates is making oneself small, indicating submissiveness [32]. Moreover, fear faces of non-human great apes share morphological similarities with their human equivalent [34], and our human play scenes contained individuals showing a ‘play face’, similar to the bonobo scenes. The finding that bonobos looked longer at neutral scenes that accompanied human erotic scenes is curious, especially because this effect was very strong. Possibly, the bonobos actively avoided human erotic scenes, or there was something about the neutral scenes that held their attention. For instance, clothing may have provided a salient cue (e.g. owing to more variation in patterns and colours). While this is also true for the other stimulus pairs, within the erotic scenes, humans were almost fully unclothed. In hindsight, a better match for the erotic scenes would have been neutral scenes displaying humans in swimwear.

Human participants showed a general bias toward emotional scenes over neutral scenes, and emotional human scenes received slightly more attention than bonobo scenes. Distress scenes elicited the strongest bias, but humans also looked longer at embracing/grooming, play and erotic/sex scenes. Yawns did not elicit a clear bias for either depicted species. In a previous study, participants rated the same human yawning scenes as least intense compared with other categories [10], which may explain the lack of a bias towards yawns. Nevertheless, the bonobo yawning scenes were previously scored as negative and intense, yet did not elicit a bias in our study. We have no clear explanation for this discrepancy, but yawns may be more ambiguous and context dependent [57] than the other categories, and therefore do not stand out among the paired social, emotionally neutral scenes.

Except for distress, the attentional pattern for emotional human scenes was similar to that for bonobo scenes: participants looked longer at affiliative scenes (grooming, play) and sex scenes of bonobos compared with neutral scenes. Additionally, we found robust evidence that humans looked away from bonobo distress scenes and toward their neutral counterparts; the opposite from what we found for distress scenes of humans. In a previous study, adults rated the same bonobo distress scenes as negative and highly arousing, possibly owing to canine visibility [10]. Participants may therefore have looked away from distress scenes. However, it remains unclear why they did not look away from yawning scenes, given that these were similarly rated in terms of arousal [10]. To date, limited work has examined how humans view emotional expressions of primates (see [10,26,62]). Here, future work could benefit from including questionnaires that measure participants’ interpretation of and feelings toward the stimuli, and physiological measures to assess emotional responses. Overall, humans have a more pronounced attentional bias toward emotions than bonobos in our study. Possibly, because humans have evolved exceptionally distinctive and exaggerated communicative faces to facilitate communication [32], they also have a sensitivity to a wider range of expressions.

### (c) Limitations and conclusions

We report several limitations to our study. First, we used static images. Dynamic emotional facial expressions provide richer information than static expressions, causing stronger activation in emotion recognition regions in the brain [63]. Nevertheless, we showed complex social and emotional scenes rather than isolated facial expressions, potentially providing more context. Alternatively, by providing this context, we potentially increased stimulus complexity, making them more ambiguous [64]. A combination of these two interpretations may explain our bonobo results in that they potentially underrepresent the interest bonobos have in emotionally salient information. Importantly, however, humans did show an emotional bias across most categories and with a similarly prepared stimulus set. Moreover, in a follow-up study with bonobos where we distinguished between expressions in the face and body, attentional priority was initially observed and sustained toward neutral faces rather than emotional faces (Y. Kim , E. Van Berlo, T. Bionda, M.E. Kret, 2024, in preparation). Currently, we do not know how bonobos *interpret* images and whether scenes provide more context than isolated faces. Future research could use dynamic emotional signals using videos or a combination of images with sound, as this has successfully revealed a bias toward aggression in chimpanzees [25].

Another limitation is our small, female-only bonobo sample size. Bonobos are rarely found in zoos and even fewer are accessible for research. Therefore, we cannot extrapolate our findings to the entire species. Nevertheless, our results converge with a small but growing body of studies indicating that emotions modulate attention in bonobos [11,12,14,17].

Perceiving emotions in others is at the foundation of more complex socio-cognitive abilities such as cooperation and empathy [1]. Here, we took a first look at early and later attentional processes of humans and bonobos viewing emotional and neutral scenes to gain more insight into the shared underlying attentional mechanisms. We add to the existing literature by showing that emotional scenes can modulate attention at early and later stages in bonobos and humans in a free-viewing task, even when a competing social, but emotionally neutral stimulus is present. We add to the existing bonobo literature by showing the first evidence of an immediate attention bias *away* from bonobo distress scenes. However, the bonobos did subsequently attend longer to distressing scenes and sexual scenes, indicating that these scenes were highly salient to them. Moreover, our findings suggest that humans have a more general bias toward emotions, both at the early and later stages of attentional processing and irrespective of the species shown, in line with our species’ extensive empathetic abilities.

## Data Availability

Data and analysis scripts are available via DataverseNL [65]. Supplementary material is available online [66].

## References

[1] Ferretti V , Papaleo F . 2019 Understanding others: emotion recognition in humans and other animals. Genes Brain Behav. **18** , 1–12. (10.1111/gbb.12544) 30549185

[2] Anderson DJ , Adolphs R . 2014 A framework for studying emotions across species. Cell **157** , 187–200. (10.1016/j.cell.2014.03.003) 24679535 PMC4098837

[3] Panksepp J . 2011 The basic emotional circuits of mammalian brains: do animals have affective lives. Neurosci. Biobehav. Rev. **35** , 1791–1804. (10.1016/j.neubiorev.2011.08.003) 21872619

[4] Darwin C . 1872 The expression of the emotions in man and animals. London, UK: John Murray. (10.1037/10001-000)

[5] Dukes D *et al* . 2021 The rise of affectivism. Nat. Hum. Behav. **5** , 816–820. (10.1038/s41562-021-01130-8) 34112980 PMC8319089

[6] Yiend J . 2010 The effects of emotion on attention: a review of attentional processing of emotional information. Cogn. Emot. **24** , 3–47. (10.1080/02699930903205698)

[7] Treue S . 2003 Visual attention: the where, what, how and why of saliency. Curr. Opin. Neurobiol. **13** , 428–432. (10.1016/s0959-4388(03)00105-3) 12965289

[8] Petersen SE , Posner MI . 2012 The attention system of the human brain: 20 years after. Annu. Rev. Neurosci. **35** , 73–89. (10.1146/annurev-neuro-062111-150525) 22524787 PMC3413263

[9] Kret ME , Roelofs K , Stekelenburg JJ , de Gelder B . 2013 Emotional signals from faces, bodies and scenes influence observers’ face expressions, fixations and pupil-size. Front. Hum. Neurosci. **7** , 1–9. (10.3389/fnhum.2013.00810) 24391567 PMC3866922

[10] Kret ME , van Berlo E . 2021 Attentional bias in humans toward human and bonobo expressions of emotion. Evol. Psychol. **19** , 14747049211032816. (10.1177/14747049211032816) 34318723 PMC10358346

[11] Kret ME , Jaasma L , Bionda T , Wijnen JG . 2016 Bonobos (Pan paniscus) show an attentional bias toward conspecifics’ emotions. Proc. Natl Acad. Sci. USA **113** , 3761–3766. (10.1073/pnas.1522060113) 26976586 PMC4833271

[12] van Berlo E , Bionda T , Kret ME . 2023 Attention toward emotions is modulated by familiarity with the expressor: a comparison between bonobos and humans. Emotion **23** , 1904–1917. (10.1037/emo0000882) 36595387

[13] Bethell EJ , Holmes A , Maclarnon A , Semple S . 2012 Evidence that emotion mediates social attention in Rhesus macaques. PLoS One **7** , e44387. (10.1371/journal.pone.0044387) 22952968 PMC3431396

[14] Kano F , Hirata S , Call J . 2015 Social attention in the two species of Pan: bonobos make more eye contact than chimpanzees. PLoS One **10** , 1–14. (10.1371/journal.pone.0129684) PMC4468221 26075710

[15] Lacreuse A , Schatz K , Strazzullo S , King HM , Ready R . 2013 Attentional biases and memory for emotional stimuli in men and male rhesus monkeys. Anim. Cogn. **16** , 861–871. (10.1007/s10071-013-0618-y) 23463380

[16] Parr LA , Modi M , Siebert E , Young LJ . 2013 Intranasal oxytocin selectively attenuates rhesus monkeys’ attention to negative facial expressions. Psychoneuroendocrinology **38** , 1748–1756. (10.1016/j.psyneuen.2013.02.011) 23490074 PMC3743934

[17] Laméris DW , Verspeek J , Eens M , Stevens JMG . 2022 Social and nonsocial stimuli alter the performance of bonobos during a pictorial emotional Stroop task. Am. J. Primatol. **84** , e23356. (10.1002/ajp.23356) 34985806

[18] Allritz M , Call J , Borkenau P . 2016 How chimpanzees (Pan troglodytes) perform in a modified emotional Stroop task. Anim. Cogn. **19** , 435–449. (10.1007/s10071-015-0944-3) 26613593

[19] Hopper LM , Allritz M , Egelkamp CL , Huskisson SM , Jacobson SL , Leinwand JG , Ross SR . 2021 A comparative perspective on three primate species’ responses to a pictorial emotional Stroop task. Animals (Basel) **11** , 588. (10.3390/ani11030588) 33668170 PMC7995981

[20] van Rooijen R , Ploeger A , Kret ME . 2017 The dot-probe task to measure emotional attention: a suitable measure in comparative studies? Psychon. Bull. Rev. **24** , 1686–1717. (10.3758/s13423-016-1224-1) 28092078

[21] Wilson DA , Tomonaga M . 2018 Exploring attentional bias towards threatening faces in chimpanzees using the dotprobe task. PLoS One **13** , 1–17. (10.1371/journal.pone.0207378) PMC6261591 30485317

[22] Kret ME , Muramatsu A , Matsuzawa T . 2018 Emotion processing across and within species: a comparison between humans (Homo sapiens) and chimpanzees (Pan troglodytes). J. Comp. Psychol. **132** , 395–409. (10.1037/com0000108) 30024235

[23] Laméris DW , van Berlo E , Roth TS , Kret ME . 2022 No evidence for biased attention towards emotional scenes in Bornean orangutans (Pongo pygmaeus). Affect. Sci. **3** , 772–782. (10.1007/s42761-022-00158-x) 36519144 PMC9743850

[24] Leinwand JG , Fidino M , Ross SR , Hopper LM . 2022 Familiarity mediates apes’ attentional biases toward human faces. Proc. R. Soc. B. **289** , 20212599. (10.1098/rspb.2021.2599) PMC9043736 35473378

[25] Kano F , Tomonaga M . 2010 Attention to emotional scenes including whole-body expressions in chimpanzees (Pan troglodytes). J. Comp. Psychol. **124** , 287–294. (10.1037/a0019146) 20695660

[26] Pritsch C , Telkemeyer S , Mühlenbeck C , Liebal K . 2017 Perception of facial expressions reveals selective affect-biased attention in humans and orangutans. Sci. Rep. **7** , 1–12. (10.1038/s41598-017-07563-4) 28798378 PMC5552869

[27] Crump A , Arnott G , Bethell EJ . 2018 Affect-driven attention biases as animal welfare indicators: review and methods. Animals **8** , 136. (10.3390/ani8080136) 30087230 PMC6115853

[28] Lewis LS , Krupenye C . 2022 Eye-tracking as a window into primate social cognition. Am. J. Primatol. **84** , e23393. (10.1002/ajp.23393) 35635515

[29] Hopper LM , Gulli RA , Howard LH , Kano F , Krupenye C , Ryan AM , Paukner A . 2021 The application of noninvasive, restraint-free eye-tracking methods for use with nonhuman primates. Behav. Res. Methods. **53** , 1003–1030. (10.3758/s13428-020-01465-6) 32935327

[30] Issa HA , Staes N , Diggs-Galligan S , Stimpson CD , Gendron-Fitzpatrick A , Taglialatela JP , Hof PR , Hopkins WD , Sherwood CC . 2019 Comparison of bonobo and chimpanzee brain microstructure reveals differences in socio-emotional circuits. Brain Struct. Funct. **224** , 239–251. (10.1007/s00429-018-1751-9) 30306256

[31] Brooker JS , Webb CE , de Waal FBM , Clay Z . 2024 The expression of empathy in human’s closest relatives, bonobos and chimpanzees: current and future directions. Biol. Rev. Camb. Philos. Soc. **99** , 1556–1575. (10.1111/brv.13080) 38597291

[32] Kret ME , Prochazkova E , Sterck EHMM , Clay Z , Kret ME . 2020 Emotional expressions in human and non-human great apes. Neurosci. Biobehav. Rev. **115** , 378–395. (10.1016/j.neubiorev.2020.01.027) 31991191

[33] Hecht MA , LaFrance M . 1998 License or obligation to smile: the effect of power and sex on amount and type of smiling. Pers. Soc. Psychol. Bull. **24** , 1332–1342. (10.1177/01461672982412007)

[34] Parr LA , Waller BM , Vick SJ , Bard KA . 2007 Classifying chimpanzee facial expressions using muscle action. Emotion **7** , 172–181. (10.1037/1528-3542.7.1.172) 17352572 PMC2826116

[35] van Berlo E , Díaz-Loyo AP , Juárez-Mora OE , Kret ME , Massen JJM . 2020 Experimental evidence for yawn contagion in orangutans (Pongo pygmaeus). Sci. Rep. **10** , 1–11. (10.1038/s41598-020-79160-x) 33335177 PMC7747555

[36] Palagi E , Norscia I , Demuru E . 2014 Yawn contagion in humans and bonobos: emotional affinity matters more than species. PeerJ **2** , 1–17. (10.7717/peerj.519) PMC4137654 25165630

[37] Massen JJM , Church AM , Gallup AC . 2015 Auditory contagious yawning in humans: an investigation into affiliation and status effects. Front. Psychol. **6** , 1–8. (10.3389/fpsyg.2015.01735) 26617557 PMC4636538

[38] Norscia I , Zanoli A , Gamba M , Palagi E . 2020 Auditory contagious yawning is highest between friends and family members: support to the emotional bias hypothesis. Front. Psychol. **11** , 442. (10.3389/fpsyg.2020.00442) 32317997 PMC7147458

[39] Pool E , Brosch T , Delplanque S , Sander D . 2016 Attentional bias for positive emotional stimuli: a meta-analytic investigation. Psychol. Bull. **142** , 79–106. (10.1037/bul0000026) 26390266

[40] Palagi E , Norscia I . 2019 The ethology of animal emotions: investigation and interpretation. Sist. Intell. **31** , 11–32. (10.1422/92933)

[41] De Waal FBM . 1988 The communicative repertoire of captive bonobos (Pan paniscus), compared to that of chimpanzees. Behaviour **106** , 183–251. (10.1163/156853988X00269)

[42] Forsell LM , Åström JA . 2012 Meanings of hugging: from greeting behavior to touching implications. Comp. Psych. **1** , 1–6. (10.2466/02.17.21.CP.1.13)

[43] Fischer B , Boch R . 1983 Saccadic eye movements after extremely short reaction times in the monkey. Brain Res. **260** , 21–26. (10.1016/0006-8993(83)90760-6) 6402272

[44] Bürkner PC . 2018 Advanced Bayesian multilevel modeling with the R package Brms. R. J. **10** , 395–411. (10.32614/RJ-2018-017)

[45] Casperd JM , Dunbar RIM . 1996 Asymmetries in the visual processing of emotional cues during agonistic interactions by Gelada Baboons. Behav. Process. **37** , 57–65. (10.1016/0376-6357(95)00075-5) 24897159

[46] Makowski D , Ben-Shachar MS , Chen SHA , Lüdecke D . 2019 Indices of effect existence and significance in the Bayesian framework. Front. Psychol. **10** , 2767. (10.3389/fpsyg.2019.02767) 31920819 PMC6914840

[47] Depaoli S , van de Schoot R . 2017 Improving transparency and replication in Bayesian statistics: the WAMBS-checklist. Psychol. Methods **22** , 240–261. (10.1037/met0000065) 26690773

[48] Heiss A . 2021 A guide to modeling proportions with Bayesian beta and zero-inflated beta regression models. Political science. See https://www.andrewheiss.com/blog/2021/11/08/beta-regression-guide/ (accessed 8 February 2024).

[49] MacLeod C , Mathews A , Tata P . 1986 Attentional bias in emotional disorders. J. Abnorm. Psychol. **95** , 15–20. (10.1037//0021-843x.95.1.15) 3700842

[50] Deaner RO , Platt ML . 2003 Reflexive social attention in monkeys and humans. Curr. Biol. **13** , 1609–1613. (10.1016/j.cub.2003.08.025) 13678591

[51] Anderson AK . 2005 Affective influences on the attentional dynamics supporting awareness. J. Exp. Psychol. Gen. **134** , 258–281. (10.1037/0096-3445.134.2.258) 15869349

[52] Shechner T , Britton JC , Pérez-Edgar K , Bar-Haim Y , Ernst M , Fox NA , Leibenluft E , Pine DS . 2012 Attention biases, anxiety, and development: toward or away from threats or rewards? Depress. Anxiety **29** , 282–294. (10.1002/da.20914) 22170764 PMC3489173

[53] Bar-Haim Y , Lamy D , Pergamin L , Bakermans-Kranenburg MJ , van IJzendoorn MH . 2007 Threat-related attentional bias in anxious and nonanxious individuals: a meta-analytic study. Psychol. Bull. **133** , 1–24. (10.1037/0033-2909.133.1.1) 17201568

[54] Miralles A , Raymond M , Lecointre G . 2019 Empathy and compassion toward other species decrease with evolutionary divergence time. Sci. Rep. **9** , 1–8. (10.1038/s41598-019-56006-9) 31862944 PMC6925286

[55] Vick SJ , Paukner A . 2010 Variation and context of yawns in captive chimpanzees (Pan troglodytes). Am. J. Primatol. **72** , 262–269. (10.1002/ajp.20781) 20014109 PMC2936766

[56] Leone A , Ferrari PF , Palagi E . 2014 Different yawns, different functions? Testing social hypotheses on spontaneous yawning in Theropithecus gelada. Sci. Rep. **4** , 1–9. (10.1038/srep04010) PMC5379258 24500137

[57] Gallup AC . 2022 The causes and consequences of yawning in animal groups. Anim. Behav. **187** , 209–219. (10.1016/j.anbehav.2022.03.011)

[58] Demuru E , Palagi E . 2012 In bonobos yawn contagion is higher among kin and friends. PLoS One **7** , e49613. (10.1371/journal.pone.0049613) 23166729 PMC3498209

[59] Tsurumi S , Kanazawa S , Yamaguchi MK . 2019 Infant brain activity in response to yawning using functional near-infrared spectroscopy. Sci. Rep. **9** , 10631. (10.1038/s41598-019-47129-0) 31337824 PMC6650597

[60] Clay Z , de Waal FBM . 2013 Bonobos respond to distress in others: consolation across the age spectrum. PLoS One **8** , e55206. (10.1371/journal.pone.0055206) 23383110 PMC3559394

[61] Samuni L , Surbeck M . 2023 Cooperation across social borders in bonobos. Science **382** , 805–809. (10.1126/science.adg0844) 37972165

[62] Maréchal L , Levy X , Meints K , Majolo B . 2017 Experience-based human perception of facial expressions in Barbary macaques (Macaca Sylvanus). PeerJ **5** , e3413. (10.7717/peerj.3413) 28584731 PMC5457665

[63] Arsalidou M , Morris D , Taylor MJ . 2011 Converging evidence for the advantage of dynamic facial expressions. Brain Topogr. **24** , 149–163. (10.1007/s10548-011-0171-4) 21350872

[64] Tottenham N , Phuong J , Flannery J , Gabard-Durnam L , Goff B . 2013 A negativity bias for ambiguous facial-expression valence during childhood: converging evidence from behavior and facial corrugator muscle responses. Emotion **13** , 92–103. (10.1037/a0029431) 22906084 PMC4006094

[65] Van Berlo E , Roth TS , Kim Y , Kret ME . 2024 Data for selective and prolonged attention to emotional scenes in humans and bonobos (10.34894/GH2Q8D)39106955

[66] van Berlo E , Roth T , Kim Y , Kret ME . 2024 Data from: Selective and prolonged attention to emotional scenes in humans and bonobos. Figshare. (10.6084/m9.figshare.c.7376111)39106955

